# Bilateral endogenous fungal endophthalmitis following elective surgical abortion: a single center case series

**DOI:** 10.1186/s12348-025-00547-w

**Published:** 2025-11-25

**Authors:** Viet Q. Chau, Jesse D. Sengillo, Flavius A. Beca, Julia L. Hudson, Thomas A. Albini, Harry W. Flynn Jr.

**Affiliations:** 1 Associated Retinal Consultants/Beaumont Health, Royal Oak, MI USA; 2 Department of Ophthalmology, Bascom Palmer Eye Institute, University of Miami, Miami, FL USA; 3 Mid-Atlantic Retina, Wills Eye Hospital, Thomas Jefferson University, Philadelphia, PA USA; 4 Associated Vitreoretinal and Uveitis Consultants, Indianapolis, IN USA; 5 Bascom Palmer Eye Institute, University of Miami School of Medicine, 900 NW 17th Street, Miami, FL 33136 USA

**Keywords:** Endogenous fungal endophthalmitis, Surgical abortion, Antifungal therapy

## Abstract

**Background:**

To describe the clinical features and management of three cases of bilateral endogenous fungal endophthalmitis following elective surgical abortion performed at the same outpatient surgical center.

**Methods:**

Case series.

**Observations:**

Three immunocompetent women presented with vision loss days to weeks following elective surgical abortions performed at a single outpatient clinic. Initial visual acuities ranged from 20/20 to 20/300. Fundoscopic examination revealed multiple white round chorioretinal lesions with vitreous infiltration bilaterally in all three patients. Vitreous tap samples were obtained for culture and broad-spectrum intravitreal antimicrobials were administered. Despite negative cultures and systemic workup, the clinical presentation was consistent with fungal endophthalmitis. All patients received systemic and intravitreal antifungal therapy, leading to gradual resolution of lesions and improvement of visual acuity to 20/25 or better in all eyes.

**Conclusions:**

This cases series represents the first reported cluster of endogenous fungal endophthalmitis following surgical abortion. Prompt recognition and treatment with intravitreal and systemic antifungals can result in favorable outcomes. The findings highlight the importance of stringent sterilization protocols in outpatient surgical settings and the need for heighted clinical suspicion in similar presentations.

## Background

Endogenous endophthalmitis is characterized by purulent inflammation of intraocular fluids and tissues resulting from hematogenous spread of pathogens. Surgical abortion-related endogenous endophthalmitis is a rare complication that has been reported in case reports and small case series [1–7]. Surgical abortion (via uterine aspiration prior to 14 weeks or dilation and evacuation after 14 weeks) may disrupt mucosal barriers, potentially introducing microorganisms into the bloodstream either through endogenous vaginal flora or contaminated surgical instrumentation [8]. Here, we report a cluster of three cases of bilateral endogenous fungal endophthalmitis occurring within a one-month period following surgical abortions at a single outpatient clinic. Although systemic workup and cultures were negative, all cases were successfully treated with local and systemic antifungal therapy.

## Findings

### Case 1

**Fig. 1:**
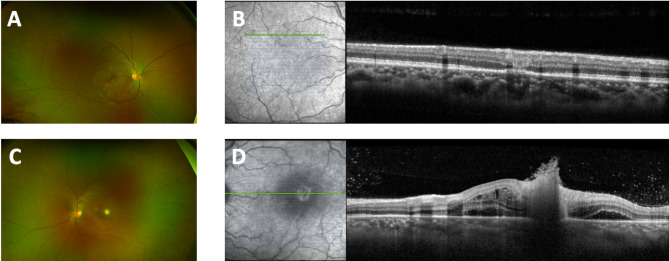
Case 1 Initial Visit (**A**) Fundus photo of the right eye with small parafoveal hypopigmented chorioretinal lesion, (**B**) En-face with OCT macula raster cross section through parafoveal hypopigmented chorioretinal lesion superiorly, (**C**) Fundus photo of the left eye with small parafoveal hypopigmented chorioretinal lesion, (**D**) En-face with OCT macula raster cross section through parafoveal hypopigmented chorioretinal lesion centrally

A 31-year-old female presented one-week post-surgical abortion with a two-day history of decreased vision, photopsia and floaters in the left eye. Visual acuity was 20/20 in the right eye and 20/300 in the left eye. Funduscopic examination revealed mild vitritis and multiple small parafoveal hypopigmented retinal lesions bilaterally, more prominent in the left eye (Fig. 1A and C). Optical coherence tomography (OCT) through these lesions confirmed macular involvement with vitreous breakthrough (Fig. 1B and D). Vitreous tap was obtained for culture and polymerase chain reaction (PCR) sequencing to rule out viral and toxoplasmosis as possible etiologies, followed by bilateral intravitreal injections of voriconazole (100 ug/0.1 mL), vancomycin (1 mg/0.1 mL), and ceftazidime (2.25 mg/0.1 mL). Systemic workup, including blood and urine cultures, CBC, metabolic panel, serum lactate, proINR, and HIV, were unremarkable. The patient was treated with systemic levofloxacin and voriconazole for 1 month, followed by voriconazole monotherapy for an additional 2 weeks. At the last follow up, 2 months after presentation, visual acuity improved to 20/25 bilaterally with complete resolution of lesions.

### Case 2

**Fig. 2:**
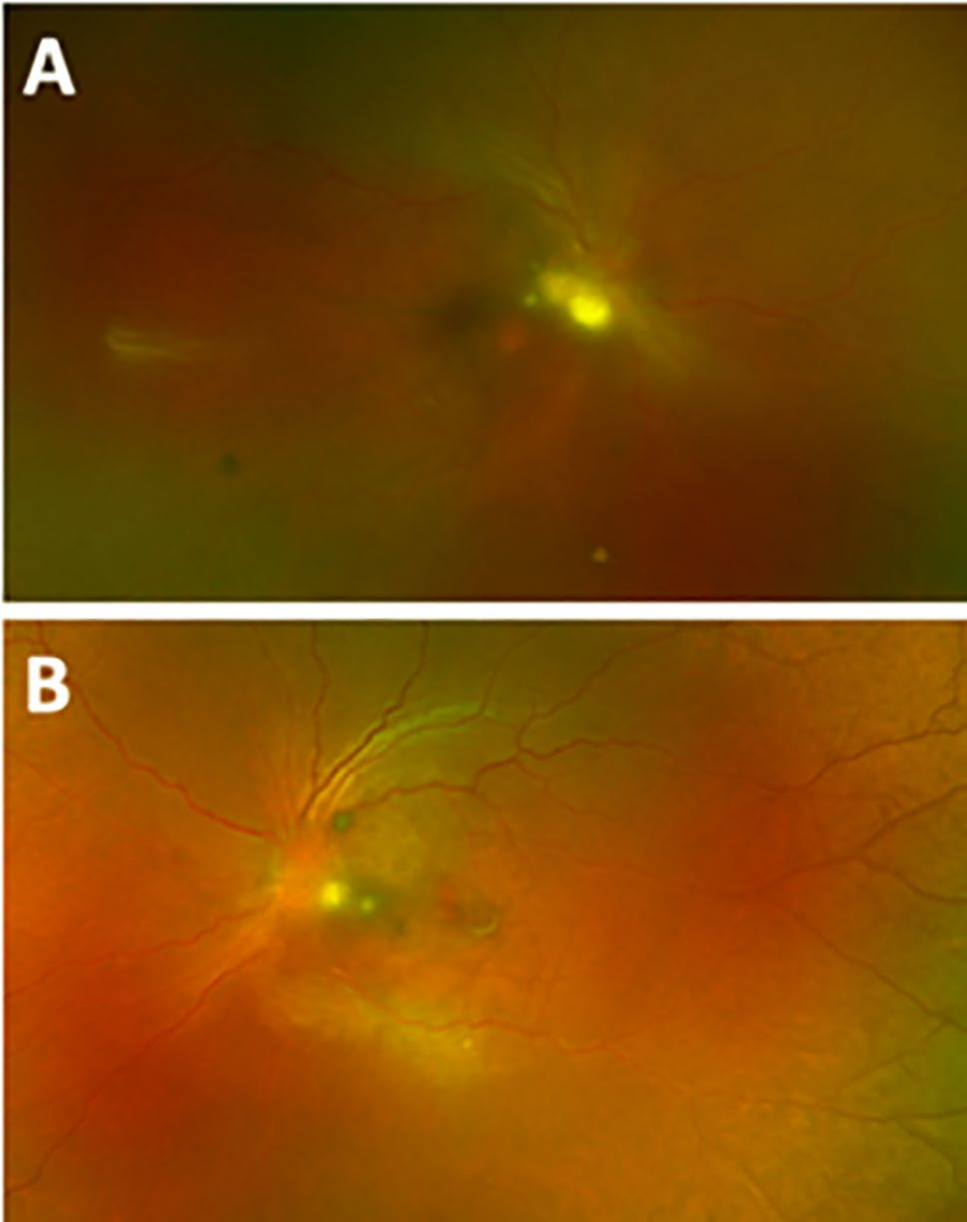
Case 2 Initial Visit (**A**) Fundus photo of the right eye with small peripapillary hypopigmented chorioretinal lesions and disc edema, (**B**) Fundus photo of the left eye with small peripapillary hypopigmented chorioretinal lesions and disc edema

A 27-year-old female developed fevers, chills and myalgias on the day of her surgical abortion, followed by bilateral floaters the next day. She presented 3 weeks later with progressive vision loss; visual acuity was 20/200 in the right eye and 20/150 in the left eye on presentation. Examination showed 2–3 + cell in the anterior chamber and 3–4 + anterior vitreous cell with moderate haze bilaterally. Bilateral grade 2 optic nerve edema was present with multifocal peripapillary infiltrates (Fig. 2A and B). Due to dense vitreous opacities, OCT was not feasible. A vitreous tap was performed in the right eye and intravitreal voriconazole (100 ug/0.1 mL), vancomycin (1 mg/0.1 mL), and ceftazidime (2.25 mg/0.1 mL) were injected bilaterally. Systemic evaluation, including blood and urine cultures, CBC, metabolic panel, serum hCG, pan CT scan, EKG, and echocardiogram, was negative. The patient received systemic voriconazole for six weeks and three additional intravitreal voriconazole injections in each eye. At the last follow up 5 months after initial presentation, vision improved to 20/25 in the right eye, and 20/15 in the left eye, with regression of chorioretinal lesions and inactive appearance of the prior vitreous fungal ball.

### Case 3

**Fig. 3:**
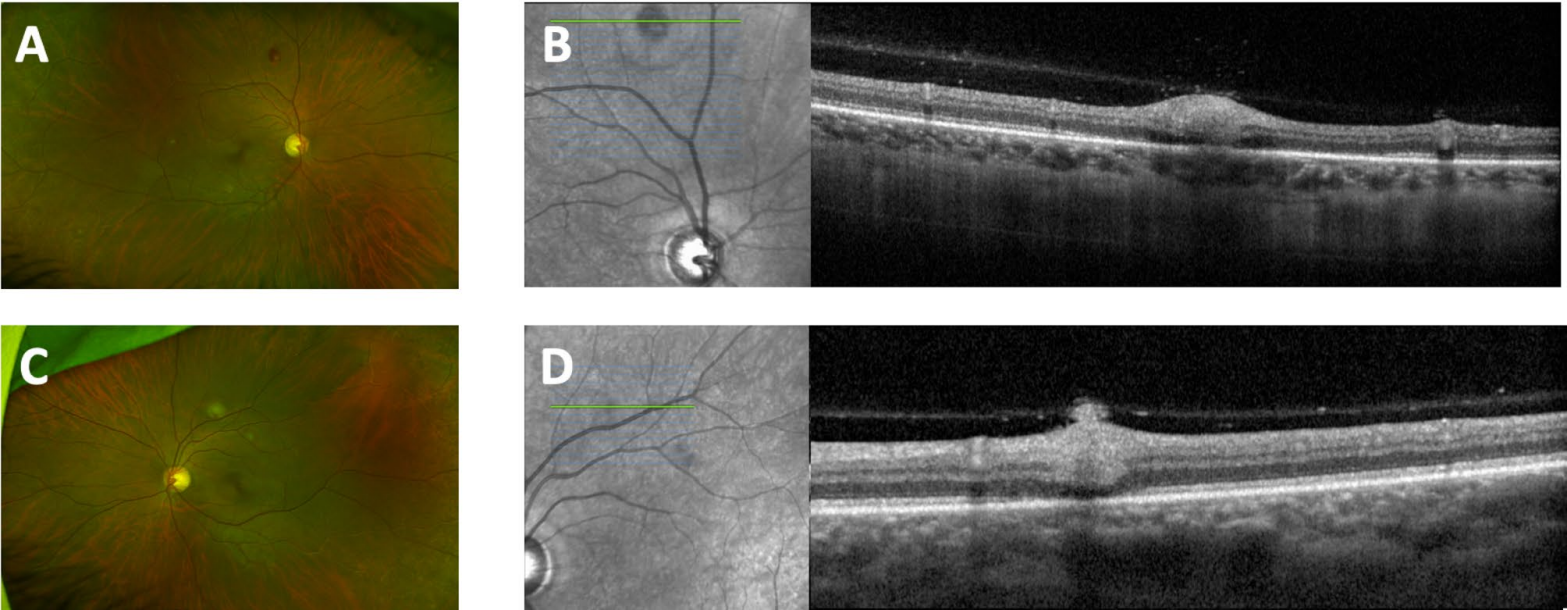
Case 3 Initial Visit (**A**) Fundus photo of the right eye with small hypopigmented chorioretinal lesions in the posterior pole and mid-periphery (**B**) En-face with OCT raster cross section through superior chorioretinal lesion, (**C**) Fundus photo of the left eye with small hypopigmented chorioretinal lesions in the posterior pole and mid-periphery, (**D**) En-face with OCT raster cross section through superior chorioretinal lesion

A 38-year-old female presented four weeks post-surgical abortion with a three-day history of photophobia and redness in both eyes. Visual acuity was 20/20 in both eyes, and exam demonstrated mild limbal injection, trace anterior chamber and anterior vitreous cell bilaterally. Fundus examination revealed scattered creamy chorioretinal lesions with variable retinal hemorrhage in both eyes (Fig. 3A and C). OCT demonstrated chorioretinal involvement and internal limiting membrane disruption (Fig. 3B and D). The patient reported fevers, chills, night sweats, and back pain. A vitreous tap of the right eye was performed followed by injection of voriconazole (100 ug/0.1 mL), vancomycin (1 mg/0.1 mL), and ceftazidime (2.25 mg/0.1 mL) in both eyes. Systemic workup was negative except for an elevated Fungitell (98, normal < 60), suggestive of systemic fungal infection. The patient was treated with oral linezolid and voriconazole for one month, with voriconazole extended to six weeks. Additional intravitreal voriconazole was delivered 3 times in the right eye and 1 time in the left eye. At final follow-up 3 months after presentation, the visual acuity was maintained at 20/20 in both eyes with regression and scarring of the chorioretinal lesions.

## Discussion

We report three cases of bilateral endogenous endophthalmitis occurring within one month following elective surgical abortion, all of which were performed at the same outpatient surgical center. Although cultures from ocular and systemic sources were negative in all patients, clinical presentation was consistent with fungal endophthalmitis. Each case was managed medically with intravitreal and systemic antimicrobials, resulting in gradual resolution of chorioretinal lesions. No patient required surgical intervention. To our knowledge, this is the first published case series to report a local cluster of endogenous endophthalmitis associated with elective surgical abortion, underscoring the importance of adherence to sterilization protocols and sound surgical technique.

The most recent United States abortion surveillance published by the Center for Disease Control and Prevention (CDC) reported over 600,000 abortions performed in 2022, in which 44% were surgical abortions [8]. Although commonly performed, endogenous endophthalmitis remains an exceptionally rare but potentially vision-threatening complication. All previously reported cases of culture-positive endogenous endophthalmitis following surgical abortion were due to *Candida albicans* [1–7], a common constituent of normal vaginal flora [9] that has been shown to grow up to two-fold during pregnancy due to hormonal changes [10]. Fungal endogenous endophthalmitis is more frequently associated with immunocompromised states or prolonged hospitalizations with indwelling devices. In this series, despite negative cultures, the diagnosis of endogenous fungal endophthalmitis was supported by characteristic clinical features, therapeutic response to antifungals, and the shared risk factor of recent surgical abortion. After recognizing an increased incidence of fungal endophthalmitis, we promptly notified the Department of Health to investigate potential unifying exposures. However, no cause of infection was identified, and no further action was taken.

Given the infrequency of endogenous endophthalmitis after surgical abortion, there is no standardized management protocol. However, punctual diagnosis, comprehensive investigations, and close monitoring are important for optimizing visual outcomes. Diagnostic workup may include vitreous or aqueous sampling for culture or PCR testing followed by delivery of broad-spectrum intravitreal antibiotics with common formulations including vancomycin and ceftazidime or amikacin and clindamycin in cases of penicillin-allergic patients [11, 12]. In suspected fungal cases, intravitreal voriconazole or amphotericin should be considered [13]. Systemic evaluation, such as blood cultures, echocardiography, and additional targeted imaging modalities (e.g., CT or MR imaging) is recommended to identify possible sources of infection. Fungitell testing, which detects (1,3)-beta-D-glucan, can be a useful adjunct in evaluating systemic fungal infection but does not replace the gold standard of a positive blood or vitreous culture. False-positives may occur in patients with chronic kidney disease, in those receiving certain antibiotics (such as beta-lactams), or even in patients with systemic bacteremia [14, 15]. In one of our patients, the Fungitell test was positive; however, given negative blood and ocular cultures, we elected to continue empiric coverage with both antifungal and antibacterial therapy. Importantly, in cases of presumed endogenous fungal endophthalmitis diagnosed on clinical grounds without culture confirmation, clinicians should maintain a suspicion for bacterial endophthalmitis and ensure that antibacterial therapy is also considered. Consultation with infectious disease specialists should be made to help guide antimicrobial treatment based on regional resistance profiles and evolving guidelines. Frequency of intravitreal antimicrobials are often at the discretion of the provider based on the patient’s clinical response. Pars plana vitrectomy may be indicated if there is extensive involvement of the vitreous refractory to medical therapy.

All patients in this series received gynecologic care at the same outpatient clinic in Miami, Florida. Due to the sensitive nature of abortion services, patients may not readily disclose such procedures unless directly queried by the clinician. In all three cases, the abortion history was not initially volunteered by the patient and was only revealed upon further inquiry. This underscores the importance of maintaining a high index of suspicion in women of childbearing age presenting with fungal endophthalmitis and creating a confidential environment to elicit a thorough gynecologic history.

In summary, endogenous endophthalmitis is a rare but serious complication of surgical abortion commonly linked to *C. albicans*, that may be managed with medical therapy if recognized early. This case series is the first known to report a localized cluster following elective surgical abortion, underscoring the need for heightened clinical suspicion and prompt antifungal therapy.

## Data Availability

No datasets were generated or analysed during the current study.
